# A role for the orphan nuclear receptor TLX in the interaction between neural precursor cells and microglia

**DOI:** 10.1042/NS20180177

**Published:** 2019-02-22

**Authors:** Danka A. Kozareva, Gerard M. Moloney, Alan E. Hoban, Valerio Rossini, Ken Nally, John F. Cryan, Yvonne M. Nolan

**Affiliations:** 1 Department of Anatomy and Neuroscience, University College Cork, Cork, Ireland; 2 APC Microbiome Ireland, University College Cork, Cork, Ireland; 3 School of Biochemistry and Cell Biology, University College Cork, Cork, Ireland

**Keywords:** cell-cell communication, chemokines, hippocampus, microglia, neurogenesis, TLX

## Abstract

Microglia are an essential component of the neurogenic niche in the adult hippocampus and are involved in the control of neural precursor cell (NPC) proliferation, differentiation and the survival and integration of newborn neurons in hippocampal circuitry. Microglial and neuronal cross-talk is mediated in part by the chemokine fractalkine/chemokine (C-X3-C motif) ligand 1 (CX3CL1) released from neurons, and its receptor CX3C chemokine receptor 1 (CX3CR1) which is expressed on microglia. A disruption in this pathway has been associated with impaired neurogenesis yet the specific molecular mechanisms by which this interaction occurs remain unclear. The orphan nuclear receptor TLX (Nr2e1; homologue of the Drosophila tailless gene) is a key regulator of hippocampal neurogenesis, and we have shown that in its absence microglia exhibit a pro-inflammatory activation phenotype. However, it is unclear whether a disturbance in CX3CL1/CX3CR1 communication mediates an impairment in TLX-related pathways which may have subsequent effects on neurogenesis. To this end, we assessed miRNA expression of up- and down-stream signalling molecules of TLX in the hippocampus of mice lacking CX3CR1. Our results demonstrate that a lack of CX3CR1 is associated with altered expression of TLX and its downstream targets in the hippocampus without significantly affecting upstream regulators of TLX. Thus, TLX may be a potential participant in neural stem cell (NSC)–microglial cross-talk and may be an important target in understanding inflammatory-associated impairments in neurogenesis.

## Introduction

Hippocampal neurogenesis, the process of generating functional new neurons from neural stem cells (NSCs), occurs throughout the lifespan in most mammalian species and plays a role in certain forms of learning, memory and in mood regulation [1]. The discussion on whether hippocampal neurogenesis actually occurs in the adult human brain has recently been renewed [59,60]. However, the hypothesis that adult-generated neurons can make important functional contributions to neural plasticity and cognition across the lifespan in humans is still widely accepted (reviewed in [61]). Microglia are an essential component of the neurogenic niche in the adult hippocampus and provide trophic support for the neurogenic process [2,62]. Specifically, microglia promote the proliferation of neural precursor cells (NPCs) as well as the survival of newly born neurons through the secretion of neurotrophic factors such as insulin-like growth factor 1 (IGF-1) and brain-derived neurotrophic factor (BDNF) [3,4], and play an important role in the pruning of apoptotic adult born neurons immediately after cell birth [5,6]. Recent evidence shows that microglia in turn can be influenced by neuronal cells [63]. However, the signalling pathways underlying NPC–microglia interaction are yet to be fully explored.

Intracellular cross-talk between neurons and microglia can occur through a variety of signalling mechanisms, one of which is through the chemokine system [7,8]. Signalling occurs between the membrane-bound ligand fractalkine, also known as fractalkine/chemokine (C-X3-C motif) ligand 1 (CX3CL1), which is constitutively expressed by neurons, and its cognate receptor CX3C chemokine receptor 1 (CX3CR1), which in the healthy brain is selectively found on microglia [9–11]. During early postnatal development, signalling between the CX3CL1/CX3CR1 pair drives synaptic pruning [12], elimination of supernumerary neurons [2], and fine-tuning of anatomical connections to ensue correct functional maturation and cell positioning [13,14]. During adulthood, CX3CL1 is expressed at particularly high levels in hippocampal neurons [11] and has been shown to stimulate the survival of NPCs *in vitro* [15]. Direct evidence for the role of CX3CL1/CX3CR1 signalling in adult hippocampal neurogenesis has recently emerged. For instance, targeted knockdown or pharmacological inhibition of CX3CR1 in adult rats resulted in a marked decrease in NPC proliferation and newborn neuron survival in the subgranular zone (SGZ) of the dentate gyrus (DG), the neurogenic niche of the hippocampus [16]. Additionally, it was demonstrated that the CX3CR1-GFP knockout (CX3CR1^KO^) mice and their heterozygote littermates exhibit decreased hippocampal neurogenesis and survival in a gene-dose-dependent manner, which was coupled with reduced synaptic plasticity and impaired performance in neurogenesis-associated tasks, such as contextual fear conditioning and learning in the Morris water maze task [17]. Employing immunohistochemistry and unbiased stereology methods, the authors showed that in the absence of CX3CR1, there was a significant decrease in the number of doublecortin (DCX+) expressing newborn neurons, a marker of neurogenesis. CX3CR1 knockout mice also exhibited reduced proliferation as evidenced by a significantly lower number of cells incorporating the thymidine analogue bromodeoxyuridine (BrdU) at 24 h post injection. Indeed, CX3CR1-deficient mice were found to present with impaired hippocampal neurogenesis, not only during adulthood, but also during late adolescence/early adulthood as well as during aging [18]. Furthermore, using the same model it has been shown that CX3CL1/CX3CR1 signalling is involved in adult hippocampal, but not olfactory bulb neurogenesis [19]. Corroborating these results, another study demonstrated that the decrease in hippocampal neurogenesis in the absence of CX3CR1 expression, was coupled with reduced dendritic complexity and delayed maturation of the newborn neurons [20]. This finding illustrates that CX3CR1/CX3CL1 has a role to play in the dendritic development of new neurons and thus in integration into the neuronal circuitry. It is thus important to recognise that a lack of CX3CR1 in the adult hippocampus has detrimental effects on multiple stages of the neurogenic process from proliferation to survival and morphological maturation [17,20,53]. It has also been shown that the reduced number of DCX+ cells observed in the SGZ of CX3CR1^KO^ mice was specific to knockout of the receptor, as mice that exhibited CX3CL1 knockout did not produce the same deficit [53]. Collectively, these data position the CX3CL1/CX3CR1 pathway as a major mediator of hippocampal neurogenesis.

The orphan nuclear receptor TLX (Nr2e1) whose expression is confined to stem cells in the neurogenic niches of the adult brain is a key intrinsic regulator of hippocampal neurogenesis [21] and it exercises this role by regulating a number of different genes and pathways. For instance, through its action as a transcriptional repressor, it regulates the expression of genes involved in multiple pathways important for the generation of neurons such as cell adhesion [22], DNA replication [23] and cell cycle [24]. It targets genes such as the cyclin-dependent kinase inhibitor cyclin-dependent kinase inhibitor 1 (p21) and the tumour suppressor gene phosphatase and tensin homologue (pten) thus promoting NSCs proliferation [22,24–26], and a member of the bone morphogenetic protein family 4 (bmp4) resulting in the inhibition of NSC astrocytic differentiation [27]. TLX can also bind to its own promoter thereby suppressing its transcriptional activity through a feedback loop, which can be antagonised by the SRY-box-containing Gene 2 (Sox2), which also binds to an upstream region of the TLX gene. What is more, it has been shown *in vitro* that TLX and Sox2 interact physically, whereby Sox2 acts as a transcriptional activator of TLX, to promote NSC maintenance and self-renewal [28]. Multiple small non-coding RNAs are differentially expressed in the hippocampus and a subgroup of them (miRNAs) has been shown to fine-tune the progression of adult hippocampal neurogenesis [64]. A number of miRNAs such as miR-9, miR-let7b and miR-378 have been shown to suppress TLX expression resulting in decreased NSC proliferation and accelerated neuronal differentiation [29–31]. In summary, TLX maintains NSCs in their proliferative state through a variety of autonomous and/or parallel pathways controlled by different genes, and disruption of these genes results in altered adult neurogenesis and brain plasticity [21].

Mice with spontaneous deletion of TLX present with impaired neurogenesis, synaptic plasticity, dendritic complexity and hippocampal-dependent behaviours during adulthood [32–35]. Interestingly, we have recently shown that a lack of TLX expression in the hippocampus resulted in microglial activation [35]. Our group has also previously demonstrated using both *in vitro* and *in vivo* approaches that an inverse relationship exists between levels of TLX and the microglial derived pro-inflammatory cytokine interleukin 1β (IL-1β) in the hippocampus [35,36]. Specifically, we found a reduction in TLX expression on hippocampal NPCs *in vitro* after administration of IL-1β [36], a dramatic increase in IL-1β in the hippocampi of TLX knockout mice [35], and a protective capacity of TLX to mitigate the negative effects of IL-1β on NPCs [37]. Given that CX3CR1^KO^ mice present with increased microglial activation and hippocampal IL-1β [17] it is possible that in the absence of TLX, intracellular communication between microglia and NPCs through CX3CL1/CX3CR1 is impaired. Thus the aim of the present study was to determine whether a disturbance in CX3CL1/CX3CR1 communication mediates an impairment in TLX-related pathways (upstream regulators and downstream targets of TLX) which may have subsequent effects on hippocampal neurogenesis.

## Materials and methods

### Animals

Two-month-old male homozygous CX3CR1-GFP mice with CX3CR1 deficiency (CX3CR1^KO^ on C57BL/6 genetic background, *n*=16) and wild-type (*n*=16) controls were group-housed under standard housing conditions (temperature: 21°C and relative humidity: 55%), with food and water available *ad libitum*. The GFP gene was knocked-in under the CX3CR1 promoter [38]. The mice were obtained from the Jackson Laboratory (B6.129P-CX3CR1*^tm1Litt^*/J; mouse strain datasheet #005582) and were generation N13F2 (backcrossed for 13 generations on C57BL/6; second filial generation was used). In order to confirm the knockout in the animals, we employed PCR and the Jackson Laboratory Protocol (Stock number: 005582) called Cx3cr1^tm1Litt^alternate1 (see https://www2.jax.org/protocolsdb/f?p=116:5:0::NO:5:P5_MASTER_PROTOCOL_ID,P5_JRS_CODE:27927,005582). Two-month-old male Nr2e1^−/−^ (TLX knockout) mice and wild-type controls (129S1/SvImJ background) were housed under standard housing conditions (temperature: 21°C and relative humidity: 55%), with food and water available *ad libitum*. Breeding pairs were kindly provided by Prof. Elizabeth Simpson, University of British Colombia. Nr2e1^−/−^ mice exhibit a spontaneous deletion of the entire TLX allele, including all nine exons. However, the deletion of TLX does not affect the transcription of neighbouring genes [65]. All experiments were conducted in accordance with the European Directive 2010/63/EU, under an authorisation issued by the Health Products Regulatory Authority Ireland and approved by the Animal Ethics Committee of University College Cork.

### BrdU administration and tissue preparation

Bromodeoxyuridine (BrdU; Sigma) was administered (4 × intraperitoneal injections over the course of 6 h at 75 μg/10 ml/kg) to the Nr2e1^−/−^ and wild-type mice. Two weeks later, these mice were killed with an intraperitoneal injection of anaesthetic (0.1 ml/kg) and transcardially perfused using 0.9% phosphate buffered saline (PBS) solution followed by 4.0% paraformaldehyde (PFA) in PBS. Brains were post-fixed overnight in 4% PFA, transferred to 30% sucrose solution and subsequently flash-frozen using liquid nitrogen. Coronal sections (40 μm) through the hippocampus were collected directly on to slides in a 1:6 series, then stored at −80°C.

### Immunohistochemistry

To determine the survival of adult-born hippocampal neurons in Nr2e1^−/−^ mice, sections were double-labelled with BrdU and the neuronal marker NeuN. Sections were washed, DNA strands denatured by incubation in 2 M HCl for 45 min at 37°C, renatured in 0.1 M sodium tetraborate (pH 8.5) and then blocked in 3% normal donkey serum (NDS; Sigma D9663) to prevent non-specific binding. Sections were incubated with anti-BrdU antibody (Abcam; AB6326; 1:250) followed by AlexaFluor594 donkey anti-rat (Abcam; AB150156; 1:500) and NeuN (Millipore; MAB377; 1:100) antibodies. Sections were then incubated with AlexaFluor488 donkey anti-mouse antibody (Abcam; AB150105; 1:500), washed and coverslipped using anti-fade medium (DAKO; S3023). The number of microglia in the hippocampi of these animals was assessed by staining for ionised calcium binding adaptor molecule 1 (Iba-1). Sections were washed, incubated in 3% NDS and then in anti-Iba-1 antibody (Wako; 019-19741; 1:1000) overnight. Sections were then incubated in AlexaFluor488 donkey anti-rabbit antibody, counterstained with DAPI (Sigma; D9642; 1:5000) and coverslipped with anti-fade mounting medium (DAKO; S3023).

### Image analysis and cell quantification

Images were obtained using an Olympus FV1000 scanning laser confocal system (Biosciences Imaging Centre, Department of Anatomy and Neuroscience, University College Cork, Ireland). Z-stack images with 1.10- or 4.4-μm step size were collected using a 10× objective (BrdU/NeuN) or 20× objective (Iba1/DAPI), respectively. The DG was imaged bilaterally on all sections. Cell quantification and area measurements were performed using the image processing software ImageJ (National Institute of Health; U.S.A.; [66]). Systematic random sampling was employed for all cell quantifications. For area quantification, the area of interest (i.e. the DG or the microglia soma) was outlined manually and its area calculated using ImageJ.

### Total RNA extraction and cDNA synthesis

Animals were killed by cervical dislocation and the hippocampus was dissected out and stored in solution that stabilises and protects cellular RNA (RNA*later*; Sigma) for 48 h at 4°C, after which the RNAlater was removed and the tissue was frozen at −80°C until subsequent use. Samples were processed according to the GenElute kit protocol (Sigma; RTN350). Total RNA yield and purity were determined using the Nanodrop System (Thermo Scientific). Synthesis of cDNA was performed using 0.5 μg of normalised total RNA from each sample using ReadyScript cDNA synthesis mix (Sigma; RDRT-25RXN).

### miRNA extraction and cDNA synthesis

Total mRNA was isolated from hippocampal samples using mirVANA miRNA Isolation Kit (Life Technologies) according to manufacturer’s instructions. Total RNA yield and quality were verified using the Nanodrop2000 spectrophotometer (Thermo Scientific, Waltham, MA, U.S.A.). RNA was reverse-transcribed to cDNA using hairpin primers specific to each miRNA gene of interest on Applied Biosystem’s GeneAmp PCR System 9700.

### Quantitative real-time-PCR and miRNA quantification

Quantitative real-time PCR (qRT-PCR) was performed on samples in duplicate and triplicate in a 96-well plate (Applied Biosystems) and captured in real time using the StepOne Plus System (Applied Biosystems). Gene expression levels were calculated as the average CT value of three replicates for each sample relative to the expression of the housekeeper gene Tfrc, and quantified using the 2^−ΔΔ*C*^^T^ method [39]. Primer sequences were: 5′-CCCAAGTATTCTCAGATATGATTTCAA-3′ (forward) and 5′-AAAGGTATCCCTCCAACCACTC-3′ (reverse) for Tfrc; 5′-CTGGGCCCTGCAGATACTC-3′ (forward) and 5′-GGTGGCATGATGGGTAACTC-3′ (reverse) for TLX (Nr2e1); 5′-AGCCCGCTTCTGCAGGA-3′ (forward) and 5′-AAAGGCTCAGAGAAGCTGCG-3′ (reverse) for bmp4; 5′-CAGGGTTTTCTCTTGCAGAAG A-3′ (forward) and 5′-ATGTCCAATCCTGGTGATGTCCG-3′ (reverse) for p21; 5′-GTGGTCTGCCAGCTAAAGGTGA-3′ (forward) and 5′-TCAGACTTTTGTAATTTGTGAATGCT-3′ (reverse) for pten; 5′-TTAACGCAAAAACCGTGATG-3′ (forward) and 5′-GAAGCGCCTAACGTACCACT-3′ (reverse) for Sox2 and 5′-AGTGTGTCGGGTGTCCATTC-3′ (forward) and 5'- GTGCAAGCAACAGAGTTGGG -3' (reverse) for CX3CR1.

qRT-PCR was performed on the small RNA-enriched samples using probes (6 carboxy fluorescein-FAM) designed by Applied Biosystems (Carlsbad, CA, U.S.A.): miR-let7b, miR-9, miR-378. qRT-PCR was carried out on the StepOnePlus PCR machine (Applied Biosystems). Samples were heated to 95°C for 10 min, and then subjected to 40 cycles of amplification by melting at 95°C and annealing at 60°C for 1 min. Experimental samples were run in technical triplicates with 1.33 μl cDNA per reaction. To check for amplicon contamination, each run also contained template free controls for each probe used. The non-coding snRNA component U6, which is highly conserved and expressed across species, was used as the endogenous control. U6 was stably expressed in all samples and differences in miRNA expression were presented as fold change from control.

### Statistical analysis

All data were analysed using SPSS statistical software (SPSS 17.0, Chicago, IL). Data were analysed by an independent-sample *t* test and an α level of 0.05 was used as criterion for statistical significance. All data are presented as mean ± S.E.M.

## Results

### Negative correlation between neurogenesis and microglial activation in the hippocampus as a result of TLX deficiency

**Figure 1:**
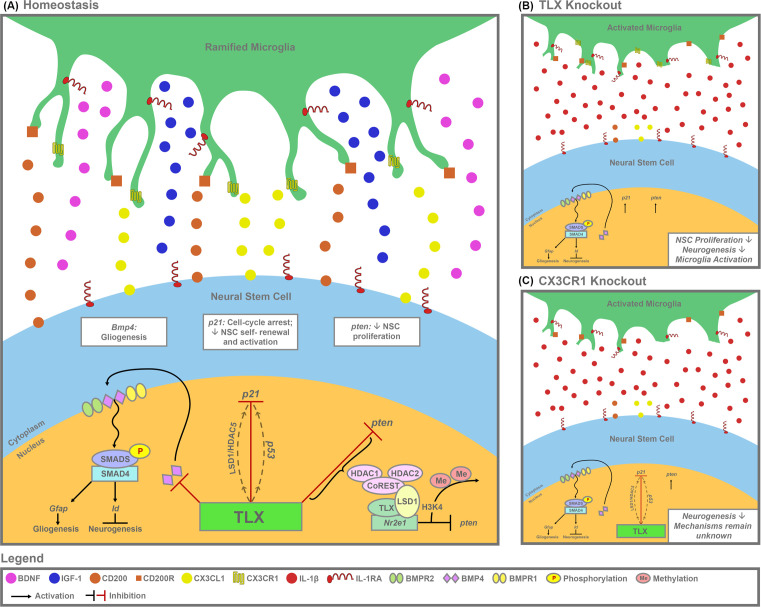
Negative correlation between neurogenesis and microglia activation in the hippocampi of TLX-deficient mice Correlation between the number of Iba1+ cells and the number of BrdU+NeuN+ cells per mm^2^ (**A**) and correlation between the soma area (μm^2^) of Iba1+ cells and the number of BrdU+NeuN+ cells (**B**) in the DG of TLX knockout (Nr2e1^−/−^) mice. Data are graphed as means (*n*=8). Representative images of coronal sections through the DG of wild-type (WT; (**C**)) and TLX knockout (Nr2e1^−/−^; (**D**)) mice immunohistochemically stained with BrdU (red) and NeuN (green). Images were taken at 10× magnification, scale bar = 100 μm. Higher magnification images depict immunopositive cells for BrdU (**C’**,**D’**), NeuN (**C’’**,**D’’**) and merged channels (**C’’’**,**D’’’**). Scale bar = 25 μm. Representative images of coronal sections through the DG of wild-type (WT; (**E**)) and TLX knockout (Nr2e1^−/−^; (**F**)) mice immunohistochemically stained with Iba1 (green) and the nuclear stain DAPI (blue). Images were taken at 20× magnification, scale bar = 50 μm.

There was a significant negative correlation between the mean number of BrdU/NeuN-positive cells and the mean number of Iba1-positive cells in the DG in mice with a spontaneous deletion of TLX (Nr2e1^−/−^; r = 0.871, *n*=8, p=0.004, Figure 1A). We found the same negative correlation between cell soma size of microglia and number of BrdU/NeuN-positive cells in the absence of TLX (Nr2e1^−/−^; r = 0.735, *n*=8, p=0.037, Figure 1B). Representative images from wild-type controls and mice with spontaneous deletion for TLX show reduced numbers of BrdU/NeuN-positive cells as well as atrophied DG morphology (Figure 1C,D), and an increased number of microglia (Figure 1E,F) in the DG of Nr2e1^−/−^ mice compared with wild-type mice.

### Confirmation of the absence of CX3CR1 in the hippocampus of CX3CR1^KO^ mice

**Figure 2:**
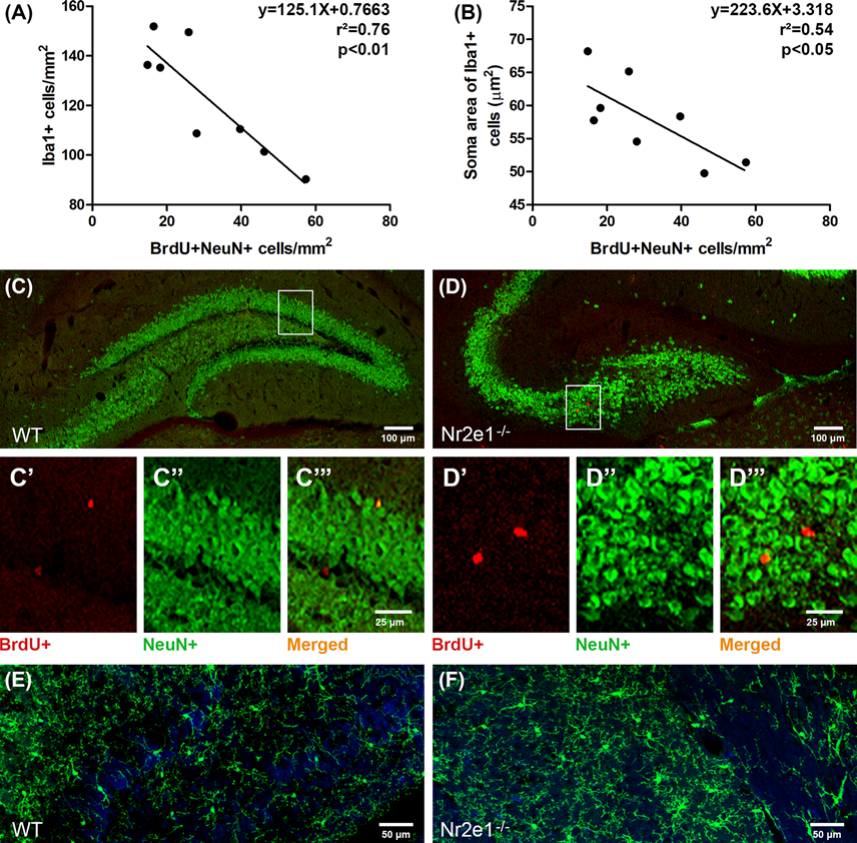
mRNA expression of CX3CR1 is detected in wild-type controls but not CX3CR1^KO^ mice Relative mRNA expression of CX3CR1 in the hippocampus of wild-type controls and CX3CR1^KO^ mice. Box and whisker plots show mean, first and third quartiles, and maximum and minimum values (*n*=8).

qRT-PCR analysis of the hippocampi of CX3CR1^KO^ and wild-type control mice confirmed the absence of the CX3CR1 gene in CX3CR1^KO^ mice (Figure 2).

### mRNA expression of TLX but not its transcription activator Sox2 is down-regulated in the hippocampus in the absence of CX3CR1

**Figure 3:**
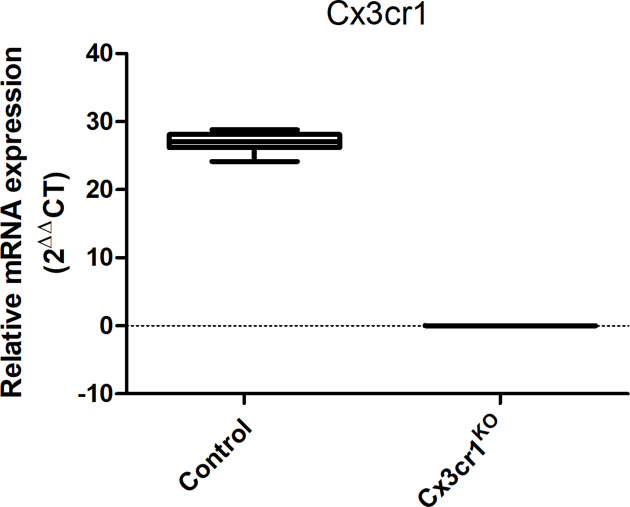
mRNA expression of TLX but not its transcription activator Sox2 is down-regulated in the hippocampus in the absence of CX3CR1 Relative mRNA expression of TLX (**A**) and Sox2 (**B**) in the hippocampus of wild-type control and CX3CR1^KO^ mice. All values were adjusted to the relative expression of the housekeeping gene Tfrc. Box and whisker plots show mean, first and third quartiles, and maximum and minimum values (*n*=8). *p≤0.05, independent-sample *t* test.

TLX gene expression was significantly decreased in the hippocampus of CX3CR1^KO^ mice compared with controls (*t* (14) = 2.115, p=0.05; Figure 3A). There was no difference in expression of the TLX regulator Sox2 between wild-type controls and CX3CR1^KO^ mice (Figure 3B).

### Lack of CX3CR1 is not associated with changes in expression of miRNAs regulating TLX in the hippocampus

**Figure 4:**
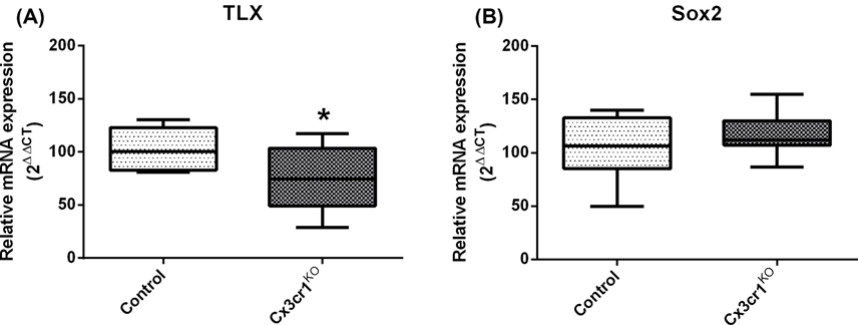
Lack of CX3CR1 is not associated with changes in expression of miRNAs regulating TLX in the hippocampus miRNA expression in wild-type controls and CX3CR1^KO^ mice for miR-let7b (**A**), miR-9 (**B**) and miR-378 (**C**). Values are expressed relative to the non-coding RNA component U6. Box and whisker plots show mean, first and third quartiles, and maximum and minimum values (*n*=8). ^+^p=0.07, independent-sample *t* test.

Upon examination of upstream miRNAs regulating TLX we found no change in expression of miR-let7b (Figure 4A) or miR-9 (Figure 4B) in hippocampal tissue between control and CX3CR1^KO^ mice. Nonetheless, we observed a trend towards increased expression of the TLX suppressor miR-378 in CX3CR1^KO^ mice compared with the wild-type controls (*t* (14) = 1.925, p=0.07; Figure 4C).

### TLX target genes bmp4 and pten, but not p21 are up-regulated in the hippocampus of CX3CR1^KO^ mice

**Figure 5:**
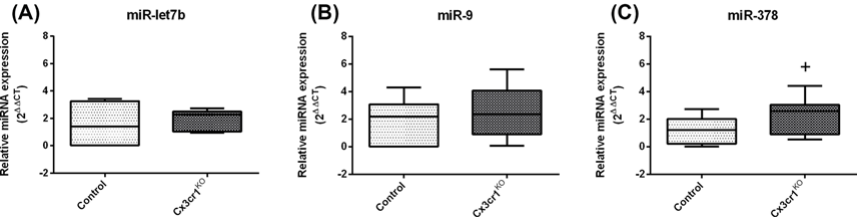
TLX target genes bmp4 and pten, but not p21 are up-regulated in the hippocampus of CX3CR1^KO^ mice Relative mRNA expression of bmp4 (**A**), pten (**B**) and p21 (**C**) in the hippocampus of wild-type control and CX3CR1^KO^ mice. All values were adjusted to the relative expression of the housekeeping gene Tfrc. Box and whisker plots show mean, first and third quartiles, and maximum and minimum values (*n*=8). *p<0.05, independent-sample *t* test. Abbreviation: bmp4, bone morphogenic protein 4.

When genes targeted by TLX were measured, we detected significantly higher relative expression of bmp4 (*t* (14) = 2.228, p=0.04; Figure 5A) and pten (*t* (14) = 2.718, p=0.02; Figure 5B) in the hippocampi of CX3CR1^KO^ mice compared with wild-type controls. There was no alteration in relative expression of the cyclin-dependent kinase inhibitor gene p21 another target of TLX, between the hippocampi of wild-type controls and the CX3CR1^KO^ mice (Figure 5C).

## Discussion

Here we have shown that in the absence of CX3CR1, TLX transcription within the hippocampus is reduced. This reduction in TLX relative mRNA abundance is not associated with changes in miR-let7b, miR-9 or miR-378, the upstream repressors of the gene, though there was a trend towards increased expression of the TLX repressor miR-378. Furthermore, no change was observed in the expression of the TLX promoter and activator Sox2. However, CX3CR1^KO^ mice exhibited an increase in bmp4 expression, a downstream target of TLX, which is involved in gliogenesis and astrocyte differentiation. In addition, in the absence of CX3CR1, hippocampal expression of the TLX repressor target pten but not p21 was increased. Collectively, these data suggest that absence of CX3CR1 promotes down-regulation of TLX expression and its downstream targets, without influencing the regulators of the gene. Thus TLX may be an important target in the cross-talk between microglia and hippocampal NSCs.

We hypothesise that the CX3CR1 signalling pathway in microglia, and TLX signalling pathways in NPCs interact to maintain homeostasis in the neurogenic niche in the adult hippocampus, which is supported by our observation of a decrease in TLX transcription in CX3CR1^KO^ mice. This is also in-line with the emerging evidence that microglia regulate neurogenesis in a temporal- and spatial-dependent manner, and that microglia are proposed as a key mediator and integrator of information that may influence the neurogenic niches (reviewed in [4]). Here we show a significant negative correlation between hippocampal neurogenesis and microglia number and soma size in the absence of TLX, which supports our previous demonstration that the absence of TLX results in an activated microglial phenotype, increased levels of endogenous IL-1β and impaired hippocampal neurogenesis [35], and thus implicate TLX as a mediator of NS/PC-microglial communication. However, the temporal relationship between the associations we observed remains unclear. Further studies examining if NPCs in the hippocampi of TLX-deficient mice have impaired CX3CL1 would provide valuable information on whether the activated microglia in TLX knockout mice has been promoted through a CX3CL1/CX3CR1-mediated pathway. Isolating microglia from the hippocampi of TLX-deficient mice and examining how these microglia behave in response to pro- and anti-inflammatory stimulation with cytokines for various durations would provide insight into the temporal relationship of our findings. Moreover, since CX3CR1-deficient mice have been shown to present with impaired hippocampal-dependent but not olfactory-dependent memory performance [17,19], it would be of interest to compare the expression of TLX and other neurogenic markers in these two neurogenic niches (the SGZ and the subventricular zone) in CX3CR1^KO^ mice. This would thus illustrate whether TLX mediates NPC–microglia cross-talk in a region-specific manner.

While it has been demonstrated that microglia have a direct effect on NSCs, the mechanisms by which microglia exercise their influence on NSCs remain largely unknown [2,4,58]. Our findings suggest that deletion of CX3CR1 expression on microglia facilitate the activation of down- but not up-stream pathways of TLX. As there was no up-regulation in mRNA expression of the transcription factor Sox2 in the hippocampi of CX3CR1^KO^ mice, it is possible that the reduction in TLX gene transcription that we observed is regulated through a self-repression mechanism. This is supported by the fact that the transcriptional regulation of NPCs by Sox2 and TLX is autonomous such that both can act independently for the potentiation of cell proliferation and the repression of cell differentiation in order to maintain the undifferentiated and self-renewable state of progenitors within the neurogenic niche [40,41].

A number of miRNAs, such as miR-9, miR-137, miR-let7d and miR-let7b, have been shown to regulate neurogenesis rate and progression through suppression of TLX *in vitro* [29,31,42–45] and are associated with suppression of TLX expression *in vivo* in hippocampi of adult mice [46]. Thus we examined whether the reduction in TLX gene transcription in adult CX3CR1^KO^ mice was associated with up-regulation of miR-let7b and/or miR-9. Our results showed no change in the expression of either miR-let7b or miR-9 in the absence of CX3CR1 in the hippocampi of knockout and wild-type mice. It has been shown that miR-let7b and miR-9 are heterochronic switch genes, which induce acceleration in NSC differentiation and reduction in their proliferation by targeting TLX expression [29,30]. Interestingly, both miRNAs not only bind to TLX, but their expression is inversely related to that of TLX such that silencing and overexpression of the miRNAs causes an inverse increase or reduction, respectively, in TLX [29,30]. This phenomenon has been demonstrated in adult hippocampal NPC cultures and in the embryonic developing brain for both miR-9 and miR-let7b [29,30] as well as in retinal progenitor cells for miR-let7b [45]. As these processes may occur autonomously in increased NPC neuronal differentiation (which would be induced by miR-let7b and/or miR-9) combined with the fact we did not observe any change in miR-let7b and/or miR-9 expression, it follows that the reduction in TLX in CX3CR1^KO^ mice is independent of either the TLX-miR-let7b regulatory loop or the TLX-miR-9 feedback pathway. This further supports our conjecture that the down-regulation of TLX observed in the absence of CX3CR1 could be a result of a self-repression mechanism, thereby positioning TLX as a potential target or co-regulator of the CX3CR1/CX3CL1 pathway.

**Figure 6:**
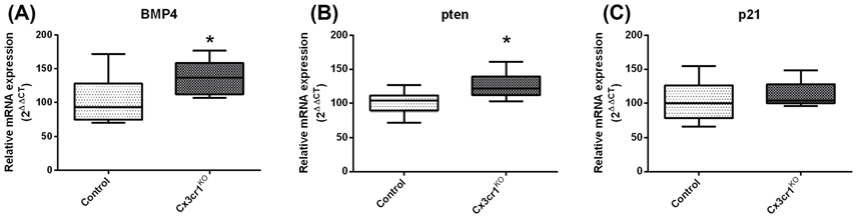
Schematic diagram illustrating possible mechanisms by which NSCs and microglia interact Under homoeostasis (**A**), TLX acts as a transcriptional repressor of a variety of genes in order to maintain NSCs in their proliferative and non-differentiative states by inhibiting gliogenesis (bmp4 pathway), cell-cycle arrest (p21 pathway) and decreased proliferation (pten pathway). Microglia send neurotrophic signals such as BDNF and IGF-1 to NSCs, and receive output from NSCs through CX3CL1/CX3CR1- and CD200/CD200R-mediated pathways. In the TLX knockout mouse (**B**), microglia become activated (retracted processes coupled with increased levels of endogenous IL-1β) and NSC proliferation and neurogenesis decreases. In the CX3CR1 knockout mouse (**C**), microglia activation and increased levels of endogenous IL-1β are coupled with reduced TLX expression, up-regulated levels of bmp4 and pten and a reduction in neurogenesis. GFAP: Glial fibrillary acidic protein; Id: Inhibitor of DNA-binding/differentiation protein; BMPR1: Bone morphogenic protein receptor, type 1; bmp4, Bone morphogenic protein 4; BMPR2: Bone morphogenic protein receptor, type 2; SMADS: homologies to the *Caenorhabditis elegans* SMA (‘small’ worm phenotype) and *Drosophila* MAD (‘Mothers Against Decapentaplegic’) family of genes; P: phosphorylation; SMAD4: Mothers against decapentaplegic homologue 4; CoREST: REST (RE1-silencing transcription factor) corepressor 1; TLX: Protein Tailless Homologue; Nr2e1: Nuclear receptor subfamily 2 Group E Member 1; Me: methylation.

miR-378 has been shown as an important enhancer of cell survival through reduction in apoptosis [47,48], reduction in cell proliferation and promotion of differentiation [31]. We observed a trend towards increased expression of miR-378 in the hippocampus of CX3CR1^KO^ mice compared with controls. Similar to miR-let7b and miR-9, miR-378 has been shown to exert its effect on NSCs through binding and suppressing the expression of TLX [31] coupled with an up-regulation of two TLX downstream targets – p21 and pten [31]. Given the trend towards increased miR-378 expression, we examined the mRNA levels of p21 and pten and observed an increase in pten but no change in p21 expression in the hippocampi of CX3CR1^KO^ mice. However, a limitation of the present study is that we examined expression levels in the whole hippocampus, rather than specifically in the DG where NSCs predominantly reside. The mechanisms by which TLX suppresses both of these genes have been extensively studied: the repression of both genes occurs through an interaction between TLX and the histone demethylase lysine-specific histone demethylase 1A (LSD1) [49,50]. In the case of pten, however, the histone deacetylases (HDAC) 1 (HDAC1) and 2 (HDAC2) are recruited to form a complex with the REST corepressor 1 (CoREST), which results in demethylation of trimethylation of histone H3 at lysine 4 (H3K4) at the proximal region of pten and hence its suppression [51] (Figure 6). p21 repression on the other hand, can result from the interaction between TLX and LSD1 with HDAC5 [22] or from tumour protein TP53 (p53)-TLX-dependent signalling [24] (Figure 6). Within NSCs, it has been shown that blocking TLX-mediated suppression of both pten and p21 resulted in a reduction in NSC proliferation [22] and an increase in quiescent hippocampal NSCs rather than an increase in differentiating NSCs, which was coupled with activation of pten and p21 signalling pathways [24]. Thus in the context of the present study, in the absence of CX3CR1 on microglia TLX expression in NPCs is reduced, which is associated with a trend towards increased expression of the TLX suppressor miR-378 and an increase in the TLX downstream target pten but not p21. Hence, it is possible that the reduction in hippocampal neurogenesis observed in CX3CR1^KO^ mice by others [17,19] results from activation of TLX-suppressing signalling pathways that inhibit activation of quiescent NSCs and maintain them in their non-proliferative state through pten signalling mechanisms. Interestingly, it has been shown that attenuation of pten increases p21 stability in cancer stem cells [55], which may explain why we observed an increase in pten only but not in p21.

TLX is also involved in the transcriptional repression of the bone morphogenic protein 4 (bmp4)-SMAD signalling pathway which activates astrogenesis [27] (Figure 6). Specifically, TLX prevents the binding of bmp ligands to their type I (BMPR1; Figure 6) and type II (BMPR2; Figure 6) receptors; blocking the activation of these receptors prevents the phosphorylation of regulatory SMADS (Mothers against decapentaplegic homologue 4) and their dimerisation with the common cofactor SMAD4 (Figure 6; [27]). Under normal physiological conditions, the SMAD complex transcriptionally activates downstream targets which promote astrogenesis (glial fibrillary acidic protein (GFAP)) and inhibits neurogenesis via suppressing inhibitors of differentiation (inhibitor of DNA-binding/differentiation proteins (Ids); Figure 6; [27]). Interestingly, in the present study CX3CR1^KO^ mice displayed an increase in relative mRNA expression of bmp4, the downstream target of TLX, responsible for astrogenesis. Thus it would be of interest to examine in future studies whether the reduction in hippocampal neurogenesis in CX3CR1^KO^ mice is coupled with an increase in hippocampal astrogenesis. This is of particular importance, given that we previously observed an increased level of endogenous IL-1β coupled with increased microglia activation in TLX knockout mice [35]. Similarly, CX3CR1-deficient mice present with increased microglia activation and increased endogenous hippocampal IL-1β [17,19]. Due to the fact that astrocytes are major producers of IL-1β in the central nervous system [56,57], they may act as the ‘middle man’ in the cascade leading to impaired neurogenesis as a result of CX3CR1 and/or TLX deficiency. Thus, studies investigating the cause of inflammation in the absence of TLX will be key to determining the relationship between TLX and CX3CL1/CX3CR1 signalling.

It has previously been shown that microglia development and adult hippocampal neurogenesis are impaired in CX3CR1^KO^ mice [17,52], but has more recently been suggested that the decrease in neurogenesis observed in the CX3CR1-deficient mice is due to pathways independent of CX3CL1 [53]. Thus understanding the precise interactions and signalling mechanisms between and within microglia and NPCs may aid our understanding of diseases associated with defective microglia–neuronal cross-talk as well as with the neuropathology of aging [54]. Here we show that CX3CR1 deficiency in the hippocampus leads to the activation of TLX-dependent pathways within NSCs that may inhibit their self-renewal and promote their adoption of an astrocytic fate. We propose that TLX is a mediator in maintaining homoeostasis between microglia and NPCs. However, future studies are needed to examine whether TLX repression leads to impaired proliferation and neurogenesis and/or gliogenesis through CX3CL1/CX3CR1-dependent mechanisms.

## Abbreviations

BMP4: bone morphogenic protein 4
BrdU: bromodeoxyuridine
CX3CL1: fractalkine/chemokine (C-X3-C motif) ligand 1
CX3CR1: chemokine receptor 1
DCX: doublecortin
DG: dentate gyrus
HDAC: histone deacetylase
Iba-1: ionised calcium binding adaptor molecule 1
IL-1β: interleukin 1β
LSD1: lysine-specific histone demethylase 1A
NDS: normal donkey serum
NPC: neural precursor cell
Nr2e1: nuclear receptor subfamily 2 group E member 1
NSC: neural stem cell
p21: cyclin-dependent kinase inhibitor 1
PFA: paraformaldehyde
pten: phosphatase and tensin homologue
SGZ: subgranular zone
Sox2: sex-determining region Y- box2
TLX: protein tailless homologue
